# The America I Know Could Use a Good Cry: Impact of State and Civilian-Perpetrated Racial Violence on Cognitive Health

**DOI:** 10.1093/geroni/igaf122.3423

**Published:** 2025-12-31

**Authors:** Muriel Taks Calle, Betselot Wondimu, Cailyn Clemons, Paris Adkins-Jackson

**Affiliations:** Columbia University Mailman School of Public Health, New York, New York, United States; Columbia University Mailman School of Public Health, New York, New York, United States; Columbia University Mailman School of Public Health, New York, New York, United States; Mailman School of Public Health, Columbia University, New York, New York, United States

## Abstract

From a life course approach and relying on the Weathering Hypothesis as a primary pathway, this study acknowledges that cumulative stressful and fear-related experiences, whether endured directly or indirectly, have a detrimental impact on the individual’s health. Prior investigations show that exposure to the American criminal justice system experienced by individuals racialized as Black correlates with adverse cognitive effects. This analysis seeks to highlight not only the role of governmental and institutional bodies in perpetuating structural racism but also the contributions of civilian actors to racial terror which in turn, increases the chances of cognitive impairment among those racialized as Black. We estimate the association between living in a county with a history of structural racism and racially disproportionate incarceration rates (in the decade preceding the passing of the 1994 Crime Bill) and having at least one incident of cognitive impairment (2008-2020), based on cognitive test performance scores from the Health and Retirement Study (HRS). Binomial logistic regression results suggest that participants racialized as Black in counties with high structural racism (lynching history, and disproportionate deaths due to police violence) and disproportionate incarceration have 21.3 times higher odds (β = 3.059; 95%CI [0.242, 5.875]) of having at least one incident of cognitive impairment compared to those racialized as Black in counties with the same conditions except disproportionate incarceration.

